# The Cost of Blindness in the Republic of Ireland 2010–2020

**DOI:** 10.1155/2016/4691276

**Published:** 2016-02-15

**Authors:** D. Green, G. Ducorroy, E. McElnea, A. Naughton, A. Skelly, C. O'Neill, D. Kenny, D. Keegan

**Affiliations:** ^1^Novartis Ireland, Merrion Road, Dublin, Ireland; ^2^J. E. Cairnes School of Business and Economics, National University of Ireland, Galway, Ireland; ^3^Ophthalmology Department, Mater Misericordiae University Hospital, Dublin, Ireland; ^4^National Council for the Blind of Ireland, Whitworth Road, Dublin, Ireland

## Abstract

*Aims*. To estimate the prevalence of blindness in the Republic of Ireland and the associated financial and total economic cost between 2010 and 2020.* Methods*. Estimates for the prevalence of blindness in the Republic of Ireland were based on blindness registration data from the National Council for the Blind of Ireland. Estimates for the financial and total economic cost of blindness were based on the sum of direct and indirect healthcare and nonhealthcare costs.* Results*. We estimate that there were 12,995 blind individuals in Ireland in 2010 and in 2020 there will be 17,997. We estimate that the financial and total economic costs of blindness in the Republic of Ireland in 2010 were €276.6 million and €809 million, respectively, and will increase in 2020 to €367 million and €1.1 billion, respectively.* Conclusions*. Here, ninety-eight percent of the cost of blindness is borne by the Departments of Social Protection and Finance and not by the Department of Health as might initially be expected. Cost of illness studies should play a role in public policy making as they help to quantify the indirect or “hidden” costs of disability and so help to reveal the true cost of illness.

## 1. Introduction

The World Health Organization predicts that by 2020 there will be seventy-six million blind people worldwide. The Vision 2020 Global Initiative aims to eliminate the main causes of avoidable blindness by the year 2020 by facilitating the planning, development, and implementation of sustainable national eye care programmes. If this initiative is successfully implemented, it is estimated that the number of blind individuals globally will fall to 24 million by 2020 [1].

Visual impairment may be broadly defined as a limitation in one or more functions of the eye and/or visual system. It has a major impact on the daily lives of those affected as well as considerable economic effects on these individuals, their families, support agencies, society, and the state.

Currently, the main causes of blindness in Ireland are age related macular degeneration, glaucoma, cataract, retinitis pigmentosa, and diabetic retinopathy [2]. The numbers of those registered as blind in the Republic of Ireland between 2007 and 2008 due to these five main causes are given in Table 1.

It has already been estimated that the direct healthcare cost of visual impairment to the Republic of Ireland was €2.143 billion in 2010 and could rise to €2.673 billion by 2020 [3]. In arriving at this estimate, all degrees of visual impairment, that is, mild visual impairment in which best corrected visual acuity <6/12 but >6/18 and moderate visual impairment in which best corrected visual acuity <6/18 but >6/60 as well as blindness in which best corrected visual acuity <6/60, were considered and the costs due to each “category” of visual impairment were not elaborated.

The aim of this study is to estimate the prevalence of blindness in the Republic of Ireland and estimate the financial and total economic cost that relates to blindness alone between 2010 and 2020. In this study, blindness is defined as having either a visual acuity of 6/60 or less in the better eye and/or a visual field restricted to twenty degrees or less.

The financial cost of blindness includes direct and indirect healthcare costs. The direct costs of blindness are those incurred within the healthcare system by the government and/or other payers as a result of treating blindness. A significant proportion relates to expenditure on healthcare service utilisation, the provision of equipment, drug costs, and the cost of procedures. The cost of the increased incidence of depression [4, 5] and the occurrence of injurious falls [6] in those who are blind have been found to contribute significantly to the indirect healthcare costs of blindness and so are also addressed here.

The direct nonhealthcare costs of blindness reflect the economic impacts of this condition outside of the healthcare system, on wider society. These include productivity losses from lost earnings produced by an inability to work due to blindness or lower employment for the same reason and the cost of the provision of informal care. Deadweight welfare losses are losses associated with having to raise additional tax revenue to publicly fund both healthcare services and welfare payments to the blind and the taxation revenue that is lost as a result of blindness form the indirect nonhealthcare costs of blindness.

The actual financial costs described above and an economic valuation of the “burden of disease” together define the total economic cost of blindness. The burden of disease is quantified using the economic value of disability adjusted life years, DALYs, which include healthy years of life lost due to disability and life lost due to premature death associated with blindness.

## 2. Methods

### 2.1. Prevalence of Blindness

In the Republic of Ireland, the National Council for the Blind of Ireland (NCBI) maintains a national register of “blind” people. To be eligible for registration, an individual must be identified by an ophthalmologist to have a visual acuity of 6/60 or less from their better eye on a Snellen chart and/or a binocular visual field restricted to 20 degrees or less. Registration is not mandatory and there is a history of underregistration. Kelliher et al. in 2006 found this to be 21.3% [7]. Thus, the total number of blind individuals registered with NCBI is multiplied by 1.271 (1/1 − 0.213) to adjust for underregistration.

### 2.2. Direct and Indirect Healthcare Costs

The direct costs of visual impairment and blindness in the Republic of Ireland have previously been calculated by Deloitte Access Economics (DAE) [3]. It was estimated that in 2010 the total direct costs (hospital, prescription, general ophthalmic services, and capital and noncapital expenditure costs) of treating visual impairment as a whole were €116,754,168. This was projected to increase to €127.4 million in 2015 and reach €136.8 million in 2020. The proportion of these costs that could be attributed specifically to blindness was not analysed.

DAE carried out a similar cost of illness study in Australia [8] and found that only 1.7% of direct costs resulting from all degrees of visual impairment were due to blindness. This paper assumes that the direct costs of the different degrees of visual impairment in the Republic of Ireland is the same as that of Australia. Therefore, the direct cost of blindness will be 1.7% of the total direct cost of all degrees of visual impairment as already outlined by DAE.

Depression has been found to be more prevalent in the visually impaired population than the non-visually impaired population [4, 5, 9–13], 32.5% in the former compared to 16.5% in the latter [4]. It is assumed that the rate of depression in blind individuals is equal to the rate of depression in those with mild and moderate visual impairment. Thus, the excess prevalence of depression in the blind population is 16.5%. $610.10, the cost of treating depression, is taken from an American study [14] and adjusted using purchasing power parity and inflation to 2010 euro value. This cost multiplied by the excess prevalence of depression in the blind that is 16.5% is the excess cost of depression in the blind population.

The prevalence of injurious falls is higher in those with visual impairment when compared to those without visual impairment [6, 15–19]. The total cost of injurious falls of those aged over 65 in the Republic of Ireland in 2008 was estimated to be €377.27 million [16]. As they are addressed later in this study, and to avoid the possibility of double counting, the cost of carers (€15.8 million), the cost of reduced quality of life (€53.65 million), and the cost of mortality (€135 million) associated with falls are omitted here. After these costs have been excluded, the cost of injurious falls is estimated to be €172.78 million.

According to Scuffham et al., the falls of the visually impaired account for 21% of the cost of all injurious falls [6]. However, falls caused directly by visual impairment account for 10% of the cost of falls [6]. This 10% of the cost of injurious falls represents the falls of all those visually impaired. It is assumed that the cost of falls in blind individuals is equal to the cost of falls in those with mild and moderate visual impairment. Therefore, blindness accounts for 5.78%, 6.19%, and 6.62% (the prevalence of blindness in the Republic of Ireland/the prevalence of all degrees of visual impairment in the Republic of Ireland) of the cost of falls attributable to visual impairment in the Republic of Ireland that is 10% of the total cost of injurious falls for the years 2010, 2015, and 2020 respectively.

### 2.3. Direct and Indirect Nonhealthcare Costs

Blind people are less likely to be employed compared to people with no visual impairment. Further, blind people who are employed may be less productive as a result of their disability. Productivity losses due to visual impairment will depend on the age of the person when blindness first occurs. In general, the younger the blind person the greater the impact of their sight loss on productivity [20].

Frick and Foster assumed that the proportion of productivity lost is equal to the DALY weight for blindness which is 0.600 [1] believing that although the relationship between the DALY and productivity may not be linear the use of this figure leads to a conservative estimate of the productivity losses associated with blindness.

Although Frick and Foster used the age group 15–64 in their calculations, this is altered to include only those aged 18–64 years as this likely provides a more accurate reflection of the true productivity losses in the Republic of Ireland. An equal distribution of blind individuals for the years 15, 16, 17, 18, and 19 is assumed. The sum of those aged 18 and 19 years who are blind in the Republic of Ireland that is 107 is used to calculate productivity losses instead of the total number of blind individuals between the ages of 15 and 19.

Not all of those within the cohort of 18–64 years old that are available to work choose to participate in the workforce. This may be due to the individual being unable to seek work, being unable to gain employment, or choosing not to work, due to a partner's ability to support them, for example, or because the incentive to work is not high enough. This is known as the “employment-to-population ratio.” The total number of blind individuals in the age bracket of 18–64 years is multiplied by this employment-to-population ratio to estimate the number of blind people that would participate in the workforce were they not visually impaired. This employment-to-population ratio is estimated for the general population. It is assumed that the blind cohort would have similar employment characteristics to the general public were they not blind. The employment-to-population ratio was 60.4% in 2010 according to the Central Statistics Office. The average annual earnings in 2010 (€35,905) will be used to calculate the productivity losses associated with blindness for the year 2010 and also 2015 and 2020.

There is no Republic of Ireland specific data to estimate the number of informal care hours provided to the blind and so data on the informal care hours provided by those aged over 15 years in the Republic of Ireland for all conditions is considered [3]. This information, given in Table 2, shows that, for a range of conditions, 58% of informal carers provide between 1 and 14 hours of informal care per week, 11% provide 15–28 hours of informal care per week, and so forth. This report assumes that the informal care provided to blind people is the same as the informal care provided for other conditions. The lowest number in each hours per week category is used. Therefore, it is assumed that 58% of blind people receive one hour, 11% receive 15 hours, 6% receive 19 hours, and 25% receive 43 hours of informal care per week.

To calculate the economic cost to society of the provision of informal care, Deloitte Access Economics used the methodology of van den Berg et al. [21] and used the average hourly earnings in the Republic of Ireland in 2010 which was €21.79 as the value of each hour of informal care provided. Another option is to use the minimum hourly wage for the Republic of Ireland in 2010 which was €8.65 as the value of each hour of informal care provided. Given that these estimates likely over- and underestimate the cost of informal care, respectively, €15.22, the average of these two values is the value placed on each hour of informal care provided.

The public funding of healthcare, welfare payments to the blind, and taxation revenue which is lost as a result of blindness mean that the government must increase tax revenue to reach a budget neutral position. Consequently, tax rates may be higher than they otherwise would have been. An increase in taxes on goods and services results in a loss of market efficiency. Consequently, there is an associated reduction in consumer and producer surplus which is termed the deadweight welfare loss, or excess burden of tax.

Deadweight welfare loss refers to the cost of lost efficiency due to

- the need to raise additional taxation to provide health care resources for the management of blindness;
- the need to raise additional taxation to provide social welfare payments to the blind;
- lost taxation revenue to the government due to unemployment, lower employment, and lower expenditure amongst those that are blind.

This study estimated the deadweight welfare loss associated with blindness using the same methods as those adopted by DAE [3].

### 2.4. The Burden of Disease

DALYs are a nonfinancial approach to measuring the loss of well-being and premature mortality caused by illness or disease [18]. The DALY value for a blind individual is 0.6 which estimates that blind people experience a 60% lower quality of life than a healthy individual. In their estimate of the cost of disease burden due to visual impairment in the Republic of Ireland, DAE estimated the DALYs lost for those with mild and moderate visual impairment as well as for blind individuals and applied the monetary value of a statistical life year to convert these into a financial cost [3]. The number of DALYs lost due to blindness alone in the Republic of Ireland in 2010 was estimated to be 5,588 and is estimated to increase to 7,739 years in 2020.

Largely due to the paucity of particularly Irish data, there is an unavoidable degree of uncertainty surrounding the cost of blindness in the Republic of Ireland and so a sensitivity analysis is conducted around each point estimate with a ±20% variation used on most cost components. This is in keeping with the guidelines for budget impact analysis of health technologies in Ireland [3]. The exception to this is informal care costs, where the €8.65 (minimum hourly wage in 2010; lower estimate) and €21.79 (average hourly wage in 2010; higher estimate) costs per hour of informal care provided were used as we believe that these values better predict the monetary value of informal care provided. Internationally, deadweight welfare losses are estimated to be in the range of 9, 16, and 50 cents (9%, 16%, and 50%, resp.) for every additional tax euro raised by the government. The effect of reducing the deadweight welfare loss parameter estimate to the lower bound of 9% was also examined as described by Roberts et al. in 2010 [22].

## 3. Results

As Table 3 shows, the prevalence of blindness in the Republic of Ireland in 2010 is estimated at 12,955. This figure is anticipated to increase to 17,997 by 2020. The direct, indirect, and burden of disease costs are detailed in Table 4. The value within the brackets indicates the average cost per blind individual per year. Sensitivity analysis is presented in Table 5. All costs are based on 2010 costs.

## 4. Discussion

In the Republic of Ireland, the prevalence of blindness in 2003 was estimated to be 227 cases per 100,000 adults based on NCBI register data. However, it is noted that data obtained specifically from national registers of blindness can significantly underestimate the true national prevalence of both visual impairment and blindness [7, 23, 24]. Previous UK studies have found 45–60% nonregistration rates among those eligible for partial sight or blindness registration [23, 24]. In particular, Barry and Murray found 45% of eligible patients were not appropriately registered, 72% for partial sight registration and 28% for blindness registration [25]. Kelliher et al. examined underregistration in the Republic of Ireland and found that 21% of patients at an out-patients clinic were not appropriately registered [7]. While this is lower than estimates from the aforementioned studies, it nonetheless suggests that underregistration is a concern when using register data alone to estimate prevalence of blindness in the Republic of Ireland. If the underregistration of blindness in the Republic of Ireland was similar to the 28% found in the United Kingdom [25] the prevalence of blindness in the former would be expected to increase from 25,563 in 2010 to 32,721 in 2020.

The total financial cost of blindness is estimated to be €276.6 million in 2010 and is projected to rise to €370 million in 2020. Indirect costs are the largest contributors to the financial cost of blindness.

Direct costs account for just 1.96% of the financial cost of blindness. The authors acknowledge that the assumption that blindness accounts for just 1.7% of the total direct healthcare costs associated with visual impairment might be considered unusual. Blindness is however often irreversible and a deterioration in vision to that which provides for registration as blind may imply that available treatment options have been exhausted. Conversely, those with mild or moderate impairments of their vision may still have treatment options open to them to restore vision or to prevent further vision loss.

It has already been suggested that the direct costs of blindness reduce after only one year of blindness [26].

Combined, indirect costs account for 98.04% of the financial cost of blindness. It is interesting to find that in 2010 one in every three blind individuals was of working age (18 to 64 years). This is the primary reason that the cost of lost productivity in 2010 was estimated at almost €58 million. Shamanna et al. estimate that only 20% of blind people were productive and only 25% were as productive as an “average” worker [27]. Thus, our estimations of indirect costs are conservative relative to other similar studies.

With an average cost of €11,650 per blind individual, the highest indirect cost is that of informal care. This highlights the crucial role that informal carers play in the lives of blind individuals and the burden of care such carers alleviate from the state.

Regarding the measurement of deadweight welfare loss, we are mindful of the uncertainties inherent in these calculations and acknowledge that assumptions made in such calculations may not pertain to the Republic of Ireland in 2015. Although the financial cost of blindness is projected to increase by 32.6% between 2010 and 2020 the average financial cost of blindness per patient is estimated to reduce from €21,288 in 2010 to €20,390 in 2020. This is due to two factors:

1. The percentage of blind people increases at a rate greater than the total direct medical cost estimated. Therefore, although the direct medical cost due to blindness increases, this cost is shared among more blind individuals.
2. Although the total cost of productivity losses increases, it is estimated that the proportion increase in those who are blind and aged over 65 will be higher than those of working age (18–64), meaning that the average cost of productivity loss per capita will be lower in 2020.

The cost of providing social welfare and the blind pension have not been included in the above costs as these are transfer payments, not costs. Transfer payments occur where there is a payment from one economic entity to another with no good or service produced in return for payment. In economic terms this is not categorised as a real cost that is where a good or service is produced in return for payment.

DAE estimated the average financial cost of vision loss to be €1,717 per annum in 2010 [3]. This is considerably lower than the average financial cost of blindness (€21,288) in this study. This paper estimates that blindness accounted for 71.66% of the total financial cost of visual impairment in the Republic of Ireland in 2010. This is a significant proportion considering that blind individuals represent just 5.78% of the visually impaired population.

**Figure 1:**
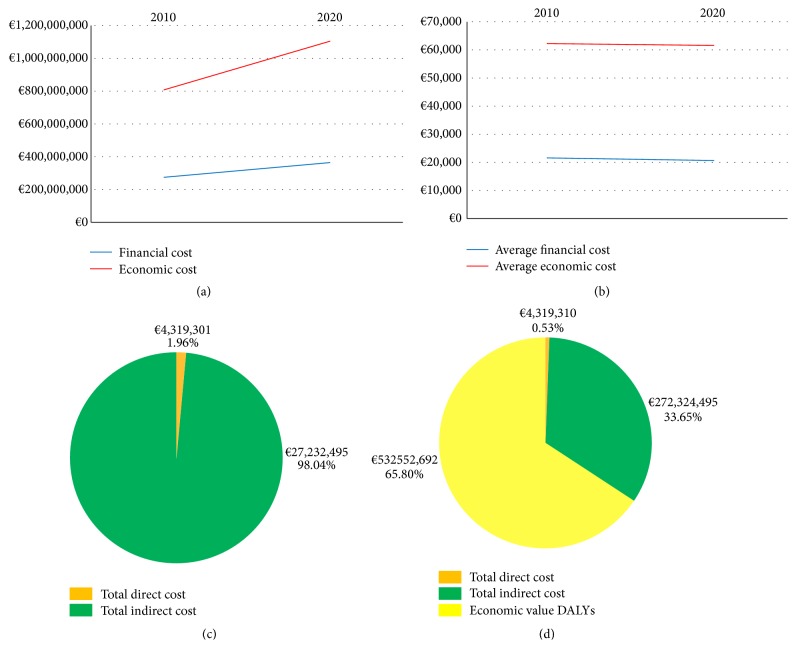
(a) The projected differential increase in the total financial and economic costs of blindness in the Republic of Ireland 2010–2020. (b) The projected differential increase in the average financial and economic costs of blindness in the Republic of Ireland 2010–2020. (c) The financial cost of blindness in the Republic of Ireland. Nonhealthcare costs account for 98.44% of the total financial cost of blindness. (d) The economic cost of blindness in the Republic of Ireland. The cost of disease burden accounts for 65.81% of the annual economic cost of blindness.

The economic cost of blindness is the sum of the financial costs of blindness and the costs associated with the burden of blindness. The total economic cost of blindness in 2010 is estimated to be €809.2 million. This is anticipated to rise to over €1.1 billion annually. The highest percentage cost of the economic cost is the disease burden of blindness which accounted for 66.8% of the total cost in 2010.  Figure 1(a) shows the rising annual financial and economic costs of blindness predicted in the Republic of Ireland.  Figure 1(b) identifies the average financial and economic costs of blindness per blind individual between 2010 and 2020.

The average cost of sight loss in the Republic of Ireland is estimated to be €9,533 compared to €62,270 for the average cost of blindness in this study. This suggests that the cost of visual impairment rises when its severity increases. This is supported by a recent study in the Netherlands where it was estimated that the annual cost of vision loss in an individual was €2,752 when the individual was mildly visually impaired but €23,331 when the individual was blind [28]. Further, these findings support the notion that interventions to prevent those with mild and moderate visual impairment from becoming blind would reduce the cost of vision loss to society in the Republic of Ireland. In an Australian study, it was found that for every dollar spent on eye care and the reduction of sight loss there could be a 4.8-fold financial return to communities [29].

## 5. Conclusion

Our knowledge and understanding of the real number of blind people must be greatly improved if we are to be effective in planning and delivering services to the people living with sight loss in the Republic of Ireland. Blindness has a significant economic impact on those directly affected by it, those who care for those directly affected, and society as a whole. The financial cost of blindness in the Republic of Ireland is estimated to be €276.6 million in 2010 and is expected to increase by 32.6% by 2020. The current estimates and projections of economic burden have significant implications for ophthalmology service requirements and the organisations providing rehabilitation and support services in Ireland in the future. It is hoped that they will form part of a comprehensive planning process for this sector of the population into the future. The direct healthcare costs of blind individuals represent just 1.96% of the total cost of blindness. Unless the indirect effects of disabilities and the costs thereof, the requirements for informal care and losses in productivity, are acknowledged and incorporated into decision making at a government level, resources will not be allocated appropriately to disabilities like blindness which may directly incur relatively low costs but exert substantial impacts on quality of life.

## Figures and Tables

**Table 1:** Numbers of those registered as blind in the Republic of Ireland according to age and category in 2007/2008.

Age range	Age related macular degeneration	Glaucoma	Cataract	Retinitis pigmentosa	Diabetic retinopathy	Total registered
45–54	12	21	26	105	36	766
55–64	38	29	38	116	55	886
65–74	138	66	35	69	82	985
75–84	745	142	41	58	80	1952
85+	1311	276	63	22	36	2955

Total registered	2244	534	203	370	289	7544

**Table 2:** The number of informal care hours provided by informal carers over the age of 15 years in the Republic of Ireland.

Hours of informal care provided per week	Reported hours of informal care received	Percentage of the blind population receiving such hours of informal care per week
1–14	1	58%
15–28	15	11%
29–42	29	6%
43+	43	25%

From the Central Statistics Office 2007.

**Table 3:** The estimated prevalence of blindness in the Republic of Ireland 2010–2020.

Age group	2010		2015		2020	
	Male	Female	Male	Female	Male	Female
0–4	43	51	48	56	49	57
5–9	80	69	89	76	98	83
10–14	144	122	156	134	172	147
15–19	142	125	150	130	161	142
20–24	163	164	150	148	153	150
25–29	189	189	166	172	147	150
30–34	214	202	247	234	213	209
35–39	240	217	279	248	316	283
40–44	239	248	279	284	320	321
45–49	259	247	288	264	334	300
50–54	273	263	309	298	341	318
55–59	300	305	333	339	376	384
60–64	347	285	381	314	424	349
65–69	339	283	432	362	478	400
70–74	383	388	463	454	597	583
75–79	483	610	563	676	699	802
80–84	628	952	780	1032	954	1170
85–89	577	1223	732	1359	981	1544
90+	603	1406	964	1883	1,442	2349

Total	5,647	7349	6,809	8,463	8,256	9741
Combined total	12,996		15,272		17,997	

**Table 4:** The average and total direct and indirect healthcare and nonhealthcare costs and burden of disease costs 2010–2020.

	2010	2015	2020
(A) Healthcare costs			
Direct healthcare costs due to blindness	€1,984,821 (€153)	€2,166,130 (€142)	€2,325,585 (€129)
Cost of depression due to blindness	€1,335,821 (€103)	€1,569,680 (€103)	€1,850,002 (€103)
Cost of injurious falls due to blindness	€998,668 (€77)	€1,069,508 (€70)	€1,143,804 (€64)
Total	€4,319,310 (€332)	€4,805,318 (€315)	€5,319,391 (€296)
(B) Nonhealthcare costs			
Lost productivity	€57,916,287 (€4,457)	€63,043,004 (€4,129)	€67,779,362 (€3,766)
Informal care	€151,391,392 (€11,650)	€177,896,717 (€11,650)	€209,665,119 (€11,650)
Deadweight loss	€63,016,816 (€4,849)	€74,059,349 (€4,850)	€84,192,487 (€4,678)
Total	€272,324,495 (€20,956)	€314,999,070 (€20,643)	€361,636,968 (€20,094)

Total financial cost of blindness, (A) + (B)	€276,643,805 (€21,289)	€319,804,388 (€20,943)	€366,956,359 (€20,390)
(C) Economic value of lost well-being	€532,552,692 (€40,981)	€626,019,576 (€40,997)	€738,350,466 (€41,026)
Total economic cost of blindness, (A) + (B) + (C)	€809,196,497 (€62,270)	€945,823,964 (€61,940)	€1,105,306,825 (€61,416)

**Table 5:** Sensitivity analysis 2010–2020. The table illustrates the upper (+20%) and lower (−20%) limits around each point estimate. For informal care costs, €21.79 (average hourly wage in 2010) is used as the higher estimate and €8.65 (minimum hourly wage in 2010) is used as the lower estimate of the cost per hour of informal care provided. The effect of reducing deadweight welfare losses to 9% gives the lower estimate for this parameter. This table thus provides a range and a mean or “likely” figure for the costs associated with blindness.

	2010	2015	2020
(A) Healthcare costs			
Direct healthcare costs due to blindness	€1,587,857 €2,381,785	€1,732,904 €2,599,356	€1,860,468 €2,790,702
Cost of depression due to blindness	€1,068,656 €1,602,985	€1,255,744 €1,883,616	€1,480,002 €2,220,002
Cost of injurious falls due to blindness	€798,934 €1,198,492	€915,943 €1,283,409	€915,043 €1,372,565
Total	€3,455,447 €5,183,262	€3,904,591 €5,766,381	€4,255,513 €6,383,269
(B) Nonhealthcare costs			
Lost productivity	€46,333,030 €69,499,544	€50,434,403 €75,651,605	€54,223,490 €81,335,234
Informal care	€86,040,623 €216,742,794	€101,104,245 €254,689,190	€119,159,217 €300,171,020
Deadweight loss	€50,413,452 €75,620,179	€59,247,479 €88,871,219	€67,353,990 €101,030,984
Total	€182,787,105 €361,862,517	€210,786,127 €419,212,014	€240,736,697 €482,537,238
Total financial costs of blindness, (A) + (B)	€186,242,552 €367,045,779	€214,690,718 €424,978,395	€244,992,210 €488,920,507
Average financial cost per blind individual (higher estimate)	€*28,245*	€*27,831*	€*27,167*
Average financial cost per blind individual (lower estimate)	€*14,332*	€*14,060*	€*13,613*
(C) Economic value of total DALYs	€426,042,153 €639,063,230	€500,815,661 €751,223,491	€590,680,373 €886,020,559

Total economic cost of blindness, (A) + (B) + (C)	€608,829,258 €1,000,925,747	€711,601,788 €1,170,435,505	€831,417,070 €1,368,557,797
Average economic cost per blind individual (higher estimate)	€*77,024*	€*76,649*	€*76,044*
Average economic cost per blind individual (lower estimate)	€*46,851*	€*46,601*	€*46,198*

## References

[1] Frick K. D., Foster A. (2003). The magnitude and cost of global blindness: an increasing problem that can be alleviated. *American Journal of Ophthalmology*.

[2] Jackson A. J., O'Brien C. (2008). *Eyes on the Future Ireland 2008: A Study into the Prevalence of Blindness and Visual Impairment*.

[3] Deloitte Access Economics (2011). *The Cost of Sight Loss: The Economic Impact of Vision Impairment and Blindness in the Republic of Ireland*.

[4] Brody B. L., Gamst A. C., Williams R. A. (2001). Depression, visual acuity, comorbidity, and disability associated with age-related macular degeneration. *Ophthalmology*.

[5] Bruce M. L., Seeman T. E., Merrill S. S., Blazer D. G. (1994). The impact of depressive symptomatology on physical disability: MacArthur studies of successful aging. *American Journal of Public Health*.

[6] Scuffham P., Chaplin S., Legood R. (2003). Incidence and costs of unintentional falls in older people in the United Kingdom. *Journal of Epidemiology and Community Health*.

[7] Kelliher C., Kenny D., O'Brien C. (2006). Trends in blind registration in the adult population of the Republic of Ireland 1996–2003. *British Journal of Ophthalmology*.

[8] Access Economics (2004). The economic impact and cost of vision loss in Australia. *Report for the Centre for Eye Research Australia and the Eye Research Australia Foundation*.

[9] Pulska T., Pahkala K., Laippala P., Kivelä S.-L. (1999). Follow up study of longstanding depression as predictor of mortality in elderly people living in the community. *British Medical Journal*.

[10] Rovner B. W., Casten R. J. (2001). Neuroticism predicts depression and disability in age-related macular degeneration. *Journal of the American Geriatrics Society*.

[11] Rovner B. W., Shmuely-Dulitzki Y. (1997). Screening for depression in low-vision elderly. *International Journal of Geriatric Psychiatry*.

[12] Rovner B. W., Zisselman P. M., Shmuely-Dulitzki Y. (1996). Depression and disability in older people with impaired vision: a follow-up study. *Journal of the American Geriatrics Society*.

[13] Tolman J., Hill R. D., Kleinschmidt J. J., Gregg C. H. (2005). Psychosocial adaptation to visual impairment and its relationship to depressive affect in older adults with age-related macular degeneration. *Gerontologist*.

[14] Javitt J. C., Zhou Z., Willke R. J. (2007). Association between vision loss and higher medical care costs in Medicare beneficiaries. Costs are greater for those with progressive vision loss. *Ophthalmology*.

[15] Carabellese C., Appollonio I., Rozzini R. (1993). Sensory impairment and quality of life in a community elderly population. *Journal of the American Geriatrics Society*.

[16] Gannon B., O'Shea E., Hudson E. (2008). Economic consequences of falls and fractures among older people. *Irish Medical Journal*.

[17] Legood R., Scuffham P., Cryer C. (2002). Are we blind to injuries in the visually impaired? A review of the literature. *Injury Prevention*.

[18] Murray C., Lopez A. (1996). *The Global Burden of Disease: A Comprehensive Assessment of Mortality and Disability from Diseases, Injuries and Risk Factors in 1990 and Projected to 2020*.

[19] Salive M. E., Guralnik J., Glynn R. J., Christen W., Wallace R. B., Ostfeld A. M. (1994). Association of visual impairment with mobility and physical function. *Journal of the American Geriatrics Society*.

[20] Lafuma A., Brezin A., Fagnani F., Mimaud V., Mesbah M., Berdeaux G. (2006). Nonmedical economic consequences attributable to visual impairment: a nation-wide approach in France. *European Journal of Health Economics*.

[21] van den Berg B., Brouwer W. B. F., Koopmanschap M. A. (2004). Economic valuation of informal care. An overview of methods and applications. *European Journal of Health Economics*.

[22] Roberts C. B., Hiratsuka Y., Yamada M. (2010). Economic cost of visual impairment in Japan. *Archives of Ophthalmology*.

[23] Robinson R., Deutsch J., Jones H. S. (1994). Unrecognised and unregistered visual impairment. *British Journal of Ophthalmology*.

[24] Bunce C., Evans J., Fraser S., Wormald R. (1998). BD8 certification of visually impaired people. *British Journal of Ophthalmology*.

[25] Barry R. J., Murray P. I. (2005). Unregistered visual impairment: is registration a failing system?. *British Journal of Ophthalmology*.

[26] Meads C., Hyde C. (2003). What is the cost of blindness?. *British Journal of Ophthalmology*.

[27] Shamanna B. R., Dandona L., Rao G. N. (1998). Economic burden of blindness in India. *Indian Journal of Ophthalmology*.

[28] Boland M. R. S., Vingerling J., Groot M., Hakkaart-van Roijen L. (2011). PSS9 the burden of age-related macular degeneration in the Netherlands. *Value in Health*.

[29] Taylor H. R., Pezzullo M. L., Nesbitt S. J., Keeffe J. E. (2007). Costs of interventions for visual impairment. *American Journal of Ophthalmology*.

